# In vitro growth (IVG) of human ovarian follicles in frozen thawed ovarian cortex tissue culture supplemented with follicular fluid under hypoxic conditions

**DOI:** 10.1007/s00404-022-06672-4

**Published:** 2022-07-24

**Authors:** Andreas Schallmoser, Rebekka Einenkel, Cara Färber, Nicole Sänger

**Affiliations:** Department of Gynecological Endocrinology and Reproductive Medicine, University Hospital of Bonn, Venusberg-Campus 1, 53127 Bonn, Germany

**Keywords:** Follicle, Ovarian tissue, Reproduction

## Abstract

**Background:**

Despite its clinical success rates, transplantation after ovarian tissue cryopreservation (OTC) remains a matter of concern. Certain cancer subtypes may lead to the transfer of malignant cells when transplantation of affected ovarian tissue is conducted. IVG and subsequent isolation of vital follicles obtained from frozen thawed ovarian tissue for further in vitro maturation (IVM) would expand current fertility protection techniques while reducing the risk of retransplanting malignant cells.

**Methods:**

A total of 216 cortical biopsies from 3 patients were included in this study in 4 treatment groups. After freezing, thawing and 8 days of hypoxic tissue culture supplemented with different concentrations of human follicular fluid (HuFF) and follicle-stimulating hormone (FSH), follicles were isolated enzymatically and stained with calcein to determine follicular viability. Numbers and size of vital follicles were assessed by fluorescence microscopy (Ti2, Nikon) and specified by computer assisted, semi-automated measurement (NIS software, Nikon). To estimate the effect of in vitro culture on apoptosis, tissue sections were stained for nicked DNA (TUNEL) prior and after tissue culture.

**Results:**

Analysing 3025 vital follicles, we observed significant differences [*P* < 0.01] regarding follicle size when hypoxic tissue culture was supplemented with HuFF compared with the control group on day 1, individual follicles reached sizes > 100 µm.

**Conclusions:**

The results implicate that HuFF contains valuable factors contributing to significant IVG of follicles in human ovarian tissue and could be regarded as an additional tool in personalized fertility restoration prior to retransplantation of ovarian tissue.

## What does this study add to the clinical work

**Table:** 

This culture approach could facilitate a higher follicular yield while isolating ovarian follicles from unstimulated ovarian tissue bears the risk of obtaining only a small proportion of follicles suited for further single follicle culture.	

## Introduction

In reproductive medicine, the area of fertility preservation has seen a rapid evolvement of techniques in the last 20 years due to the fact, that cancer treatment influences negatively on female and male fertility [1]. Seeing an increase in the incidence of cancer and rising numbers of cancer survivors [2], high attention has been paid on the relevance of gonadotoxic potential and diverse side effects of treatments of different cancer types [3, 4]. In general, female cancer patients requiring fertility preservation have the option of oocyte cryopreservation and OTC for fertility preservation [5]. When first-line chemotherapy cannot be rescheduled, OTC is the only possibility for fertility protection in children and prepubertal girls [6–8]. Previous studies have reported pregnancies and live births after transplantation of cryopreserved and thawed ovarian tissue, proofing that OTC is a safe alternative method of fertility preservation [9–17].

However, despite all its success, transplantation after OTC remains a matter of concern as ovarian tissue from certain cancer patients may contain cancer cells and transplantation of ovarian tissue could lead to the recurrence of cancer [18–26].

IVG of preantral follicles obtained from OTC would be a great advantage for female patients with cryopreserved ovarian tissue after cancer treatment and could reduce the risk of transplantation of malignant cells significantly [27–32]. It could be demonstrated that after isolation of follicles, separation from cancer cells can be obtained by the application of established laboratory protocols [33–35]. Promising results in mice show that IVG of follicles is a feasible way [36–39], while in humans, limited progress in IVG of ovarian tissue derived follicles may be due to tissue specific structural differences [40–44] and the limited access of researchers to cryopreserved human ovarian tissue [45]. The group of Telfer et al. showed that in vitro maturation from human unilaminar follicles grown in a multi-step culture system to Metaphase II oocytes is possible, but also implicating that further research is needed to focus on the optimization of a multi-step culture strategy [46], to obtain epigenetically normal oocytes [47, 48]. Proteomic based research approaches on HuFF identified over 800 components [49–62] contributing to development and maturation potential of the follicle and the oocyte [63–65], including hormones [66–68], growth factors and cytokines [69–73]. It has been shown by Molaeeghaleh et al. that cultivating mouse follicles supplemented with follicular fluid contributed to oocyte maturation and follicle growth [74]. Beside the composition of follicular fluid, it is necessary to highlight the oxygen distribution of the female reproductive tract that is considered mainly hypoxic, below atmospheric composition [75, 76]. Ovarian follicle environment is considered low oxygenic [77–79], the ovarian environment is regarded mainly as avascular [80]. Oocyte development under atmospheric oxygen composition has a harmful impact on maturation and development potential [81], and it could be demonstrated that follicle culture under hypoxic conditions contributes significantly to the follicular development potential and viability in comparison to atmospheric oxygen conditions [82].

**Fig. 1:**
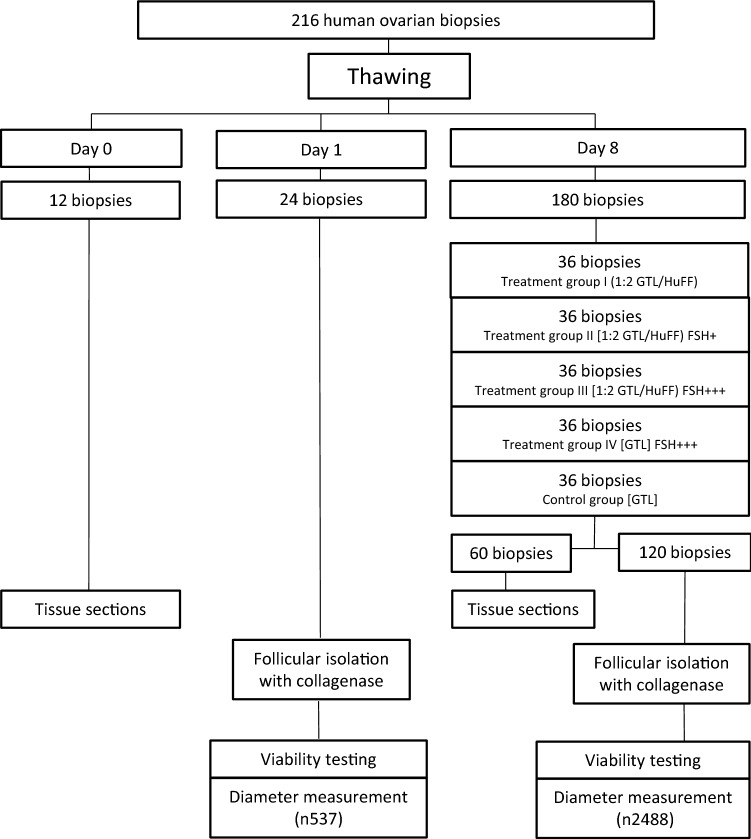
Experimental design of the study

In this prospective study, we analysed in vitro grown human follicles derived from frozen thawed ovarian cortex tissue after an 8 day period of tissue culture supplemented with human follicular fluid (HuFF) and FSH under hypoxic conditions. The experimental design of the study is indicated in Fig. 1.

## Methods

### Ethics

This study was approved by the ethics committees of the University Hospital of Bonn, Germany (007/09). Patients gave written, informed consent.

### Collection of ovarian tissue

Ovarian tissue was obtained from 3 female cancer patients prior to gonadotoxic treatment via laparoscopy. Patients donated their tissue for research purposes and were at time of cryopreservation aged 26 (diagnosed with osteosarcoma), 22 (diagnosed with astrocytoma) and 19 (diagnosed with acute lymphocytic leukaemia [ALL]) years old.

### Preparation of ovarian tissue

Cortex strips were cryopreserved and thawed according to established protocols [83–88] with minor modifications. Procedures were carried out under the European Union 2004a Directive 2004/23/EC providing maximum safety and quality of tissues [89, 90], including GMP (good manufacturing practice) procedures using certified materials and equipment under permanent hygienical and technical surveillance including particle and air germ measurement regarding room and sterile cabinet validation.

### Cryopreservation of ovarian tissue

In brief, cortex strips were equilibrated in a precooled cryopreservation solution consisting of L-15 Leibovitz’s medium (Life technologies, NY, USA) supplemented with 10% CryoSure DMSO (WAK Chemie, Steinbach, Germany), 11% human serum albumin (HSA) (Irvine Scientific, Santa Ana, CA, USA) and incubated for 35 min before slow freezing. Samples were stored in vapour phase of liquid nitrogen at −160 °C in automatically refilled storage tanks (MVE HEco Chart, Ball Ground, USA), monitored by an independent high end alarm system (Planer limited, Middlesex, GB).

### Thawing of ovarian tissue

In brief, slow frozen ovarian tissue pieces were exposed to room temperature for 40 s and submerged in a 37.5 °C pre-heated water bath for 130 s. Ovarian cortex pieces were subsequently transferred in 15 min. intervals to 3 thawing solutions consisting of DPBS CTS (Life technologies, NY, USA) supplemented with 11% human serum albumin (HSA) (Irvine Scientific, Santa Ana, CA, USA) with decreasing sucrose (Merck, Darmstadt, Germany) concentrations followed by 2 washing steps.

### Collection and processing of HuFF

HuFF was collected and pooled from 3 pre-selected fertile patients aged 25–35 years with regular cycles without gynaecological abnormalities like endometriosis or polycystic ovarian syndrome (PCO) during ovum pick up (OPU) and processed under GMP conditions to produce a batch with constant quality in composition. After sterile filtration (Acrodisc Syringe Filter, Pall, Fribourg, Switzerland) and supplementation with 50 I.U/ml penicillin and 50 μg/ml streptomycin (Merck, Darmstadt, Germany), samples were stored in liquid nitrogen [91, 92]. Concentrations of LH (luteinizing hormone), FSH (follicle-stimulating hormone), E2 (Estradiol), Prog (Progesterone), TST (Testosterone), Dehydroepiandrosterone (DHEAS) and Anti-Müllerian hormone (AMH) were determined via ELISA after processing, freezing and thawing.

### Tissue culture medium

Bicarbonate buffered, hyaluron and human serum albumin supplemented cell culture media (GTL, Vitrolife, Göteborg, Sweden) was used as a main carrier capable of maintaining a stable pH range between 7.2 and 7.4, even when supplemented 1:2 with frozen thawed HuFF. Prior to tissue culture, media were supplemented with ITS-G (Gibco, New York, USA) containing insulin (1.0 g/l), transferrin (0.55 g/l) and selenium (0.00067 g/l). Performing preliminary pH measurements in 2 day intervals over a period of 8 days, tissue culture media was incubated at 37.1 °C, 5% O_2_ and 6.8% CO_2_. Calibrator solutions (WTW, Weilheim, Germany) were incubated at 37.1 °C as well. PH measurements were performed after 2-point calibration with inolab7110 (WTW, Weilheim, Germany) using a sentix 81 pH probe (WTW, Weilheim, Germany) on day 2, 4, 6 and 8.

### Ovarian tissue culture

Thawed ovarian tissue was processed using 2 mm biopsy punches (pfm medical, Köln, Germany) to obtain 72 biopsy punches per patient. We defined 4 treatment groups and one control group per patient with different culture compositions for 8 days of tissue culture. Treatment group I (1:2 GTL/HuFF), Treatment group II (1:2 GTL/HuFF, FSH+) supplemented with 2.5 mlU/ml of recombinant FSH (Gonal-F^®^ 200 IU, Merck-Serono, Darmstadt, Germany), Treatment group III (1:2 GTL/HuFF, FSH+++) supplemented with 15 mlU/ml of recombinant FSH (Gonal-F^®^ 200 IU, Merck-Serono, Darmstadt, Germany), Treatment group IV [GTL, FSH+++] supplemented with 15 mlU/ml of recombinant FSH (Gonal-F^®^ 200 IU, Merck-Serono, Darmstadt, Germany), and a control group [GTL only]. Groups of 4 punches were cultivated in 300 µl tissue culture media under oil [GM501 Mineral Oil, Gynemed, Lensahn, Germany] at 37.1 °C and 6.8% CO_2_ respectively, to maintain a stable pH between 7.2 and 7.4 under hypoxic conditions [5% O_2_]. 12 biopsy samples per patient and group were cultured, 8/12 samples per group were processed for vitality testing and size measurement, 4/12 samples per group were used for tissue sections and staining. For tissue culture incubation, G185 flatbed incubators [K-Systems, Cooper surgical, Berlin, Germany] were used. On day 2, 100 µl of medium was removed and 200 µl of pre-gassed medium was added, increasing the droplet volume to 400 µl. On day 4 and 6, 200 µl of the media and 50% of the cell culture oil was exchanged. After thawing, day 1 control was kept in AIMV (Thermo Fisher scientific, NY, USA) medium for 24 h prior enzymatic digestion and calcein staining.

### Viability test and size measurement

On day 1 and day 8 of tissue culture, follicular viability was determined. In brief, tissue was incubated with 1 mg/ml Collagenase Type 1A (Merck, Darmstadt, Germany) supplemented with 2 µmol/l Calcein AM (Merck, Darmstadt, Germany) at 37.1 °C for 80 min shielded from light while promoting tissue resolution through cautious resuspension after 50 and 60 min. After 80 min, 120 overlapping pictures were taken via a preprogramed motorized microscope platform scanning pattern and processed for later follicle size calculation via NIS Elements software (Nikon, Düsseldorf) to determine follicle number and size of vital follicles with fluorescence microscopy (Nikon, Ti2, Düsseldorf). Vital follicles were defined as oocytes enclosed by a layer of granulosa cells (cubic or flat) with evenly distributed bright green colour. Vital cells convert the nonfluorescent calcein AM to fluorescent calcein induced by intracellular esterases after acetoxymethyl ester hydrolysis resulting in emission of green fluorescence at a wavelength of 415 nm when exposed to light of a wavelength of 495 nm [93]. Biopsy digest and viability determination procedure was carried out by two ovarian tissue experienced biologists to minimize bias through potential false identification or prolonged enzymatic exposition. Vital follicles were classified in size groups according to Kristensen et al. with minor modifications [94]. In brief, follicles were ranked in groups of  > 150 µm, > 100–150 µm, 50–100 µm and < 50 µm in relation to culture duration and media composition.

### Tissue fixation and sectioning

To complement the viability tests and size measurements, tissue sections were conducted. Biopsies were fixed in 3.7% Formalin (Carl Roth; Germany) in PBS (PAN-Biotech; Germany) at 4 °C overnight. After incubation in 15% and 30% saccharin (Merck; Germany) in PBS for 10 min each, biopsies were embedded in CryoGlue (SLEE; Germany) and frozen. Tissue was cut transversely in a cryotom (SLEE; Germany) at 5 µm. 2 separate sections of the same block containing 4 biopsies per experimental group and patient were included per Hematoxylin-and eosin (H&E)-staining.

### TUNEL staining

TUNEL Assay Kit was used following manufacturer’s description. Tissue section demasking was performed using proteinase K., for staining, slides were incubated with Br-labelled nucleotides and TdT enzyme for 1 h at 37 °C. After washing, fluorescence labelled anti-BrdU antibody was added. Microscopic observation was performed at Ti2-E. Staining control (only anti-BrdU antibody incubation) was included. Staining intensity was assessed by NTS Elements software. Tissue was marked as region of interest. Mean intensity of TUNEL staining was measured. Value of negative staining control was set at 0.

## Results

### Determination of basic hormone levels in Huff

After batch processing of pooled Huff and one cycle of cryopreservation and thawing we detected the following concentrations of LH [0.7 IU/l], FSH [4.8 mlU/ml], E2 [> 30,000 pg/ml], Prog [> 600 ng/ml], TST [3.63 ng/ml], DHEAS [2.89 µg/ml] and AMH [1.01 ng/ml], as shown in Table 1.

**Table 1:** Determination of hormonal key factors in pooled HuFF used for tissue culture

	LH [U/l]	FSH [mIU/ml]	E2 [pg/ml]	Prog [ng/ml]	TST [ng/ml]	DHEAS [µg/ml]	AMH [ng/ml]
Value	0.7	4.8	>30,000	>600	3.63	2.89	1.01

### Viability test and size measurements

Analysing the follicular viability on day 1 after thawing we classified 537 follicles as vital in the day 1 control group as indicated in Fig. 1. On day 8 after thawing, we rated 337 follicles in treatment group I, 487 follicles in treatment group II, 641 follicles in treatment group III, 680 follicles in treatment group IV and 343 follicles in the control group as vital.

**Fig. 2:**
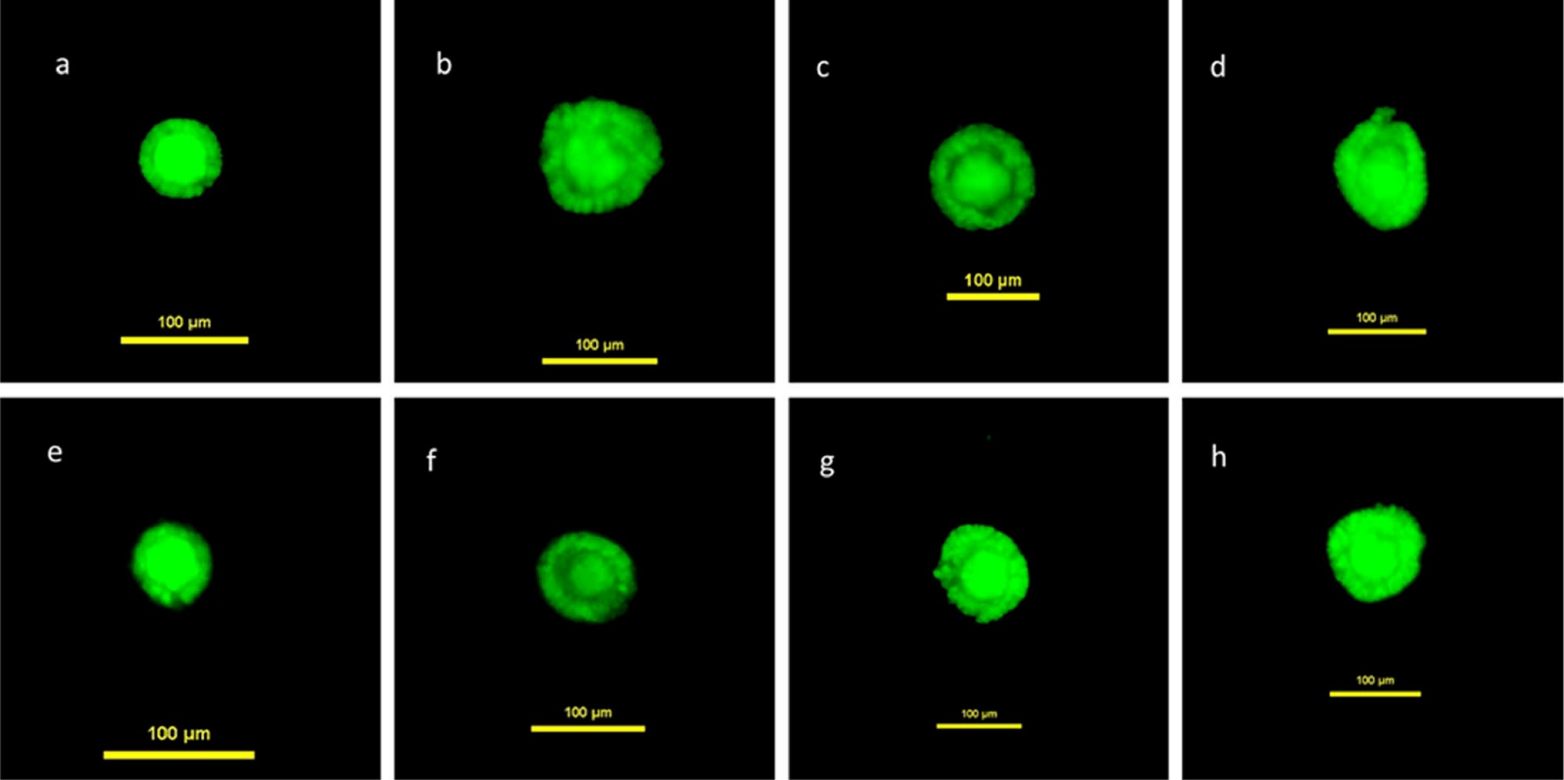
Calcein staining (green fluorescence light [495 nm]) of follicles **a**–**h**. Scalebar 100 µm

**Fig. 3:**
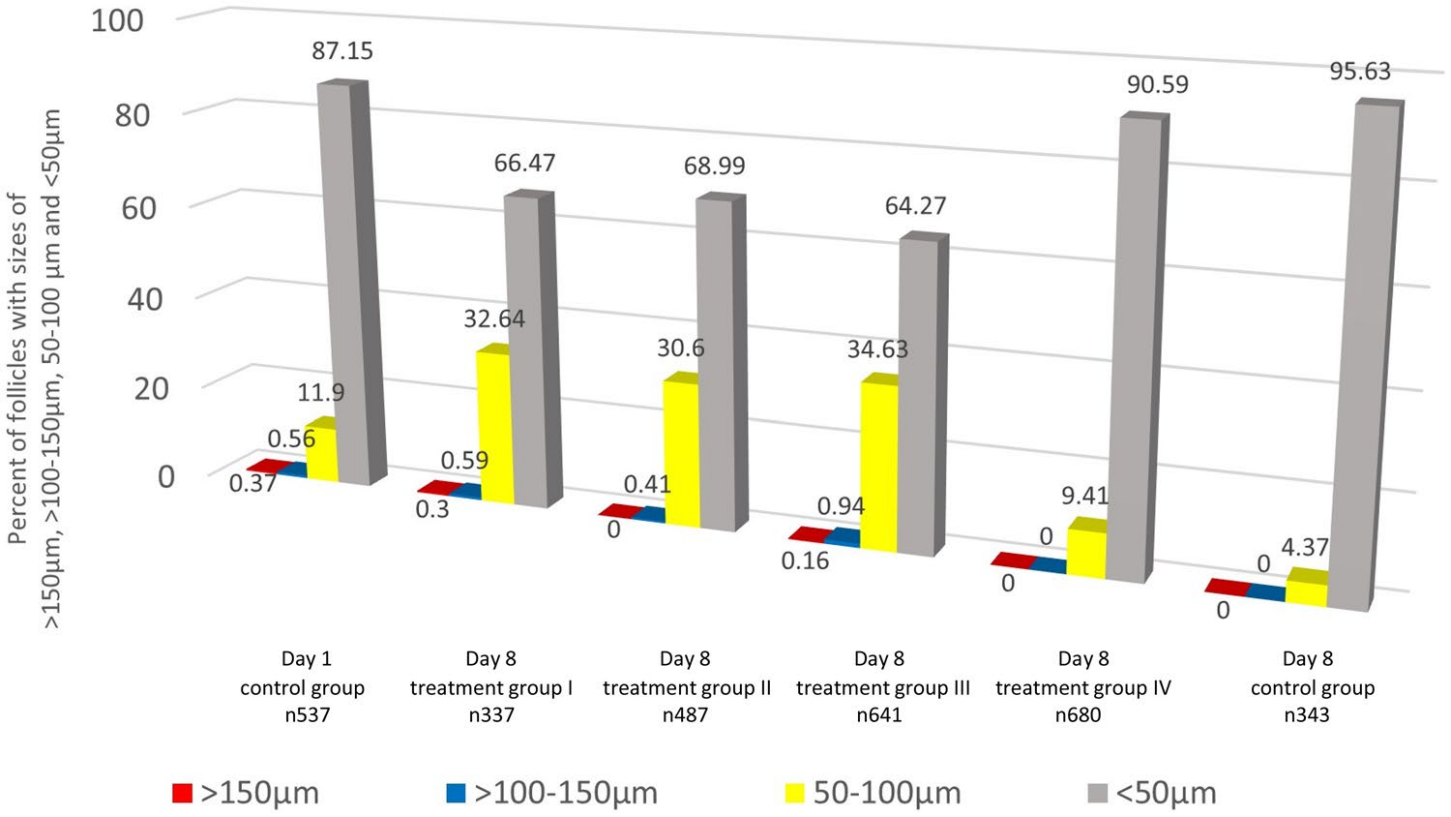
Proportion of vital follicle size [%] in relation to culture duration and media composition [*n* 3025]

**Fig. 4:**
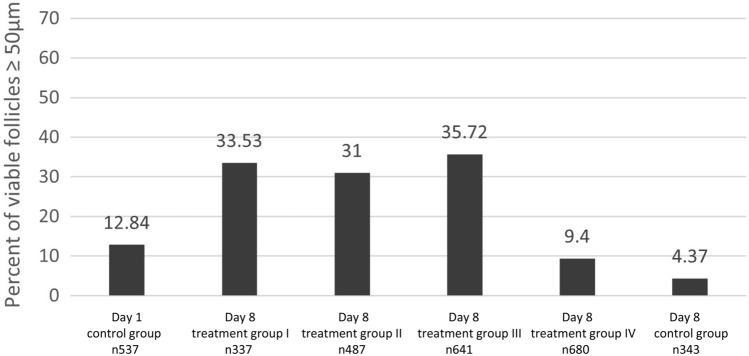
Proportion of vital follicles with size of ≥ 50 μm in relation to media composition and culture duration [*n* 3025]

**Fig. 5:**
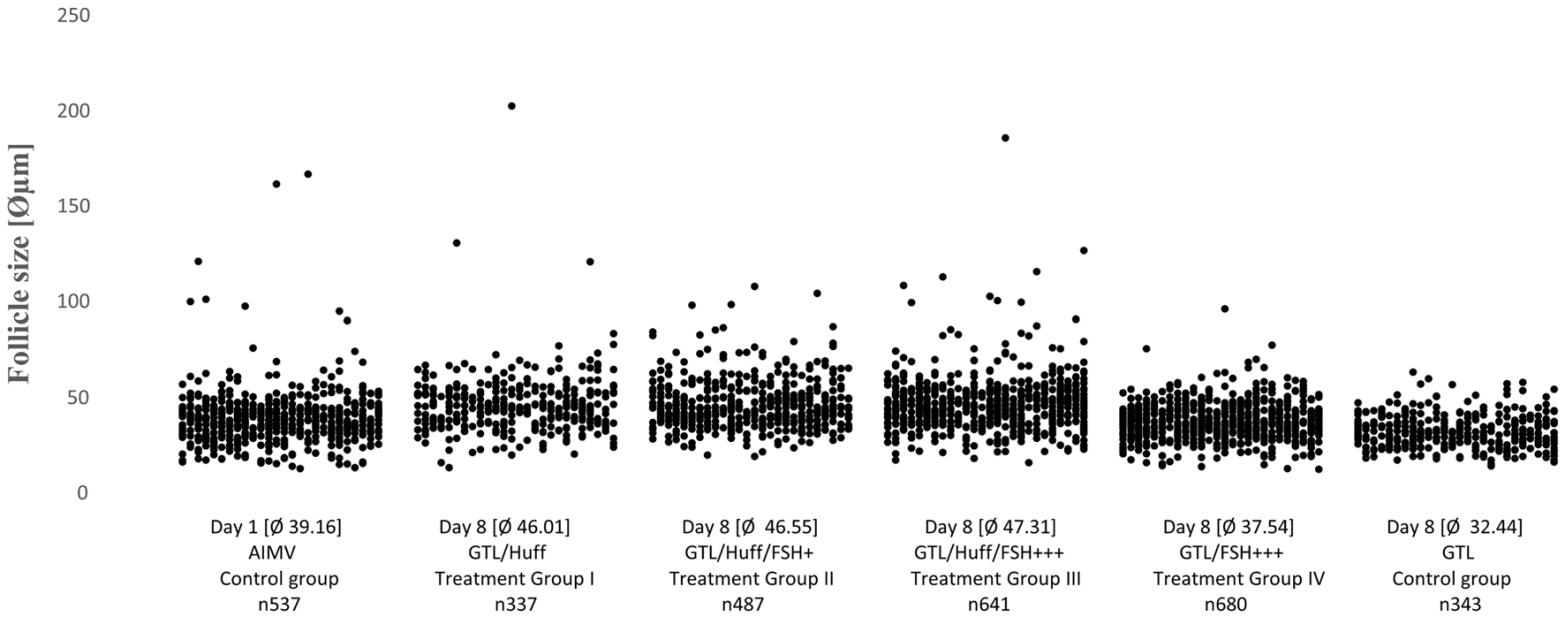
Size of vital follicles in relation to culture duration and media composition [*n * 3025]

Analysing 3025 vital follicles, our analysis showed a significant difference in vital follicle size between the control group on day 1 and treatment groups I–III on day 8 as indicated in Table 2 and Figs. 2, 3, 4, 5. In treatment groups I–III a considerable rise in numbers of vital follicles sized 50–100 µm and vitality sustainment of individual follicles sized > 100 µm were detected, results indicated in Fig. 3. In treatment group IV, vital follicle size showed a borderline significance while in the control group on day 8 the proportion of vital follicle size was significant smaller compared to the basis control on day 1. Interestingly, no vital follicles > 100 µm were detected in treatment group IV and the control group on day 8.

**Table 2:** Follicle size of study groups with different media composition and culture duration in relation to the control group on day 1

Patients 1–3 *n* 3025	Treatment group I GTL/Huff [day 8] * n* 337	Treatment group II GTL/Huff/FSH + [day 8] * n* 487	Treatment group III GTL/Huff/FSH +++ [day 8] * n* 641	Treatment group IV GTL/FSH +++ [day 8] * n* 680	Control group GTL [day 8] * n* 343
Control group [day 1] * n* 537	*P* < 0.01	*P* < 0.01	*P* < 0.01	*P* 0.095	*P* < 0.01

Mann–Whitney *U* test

### Tissue sections

**Fig. 6:**
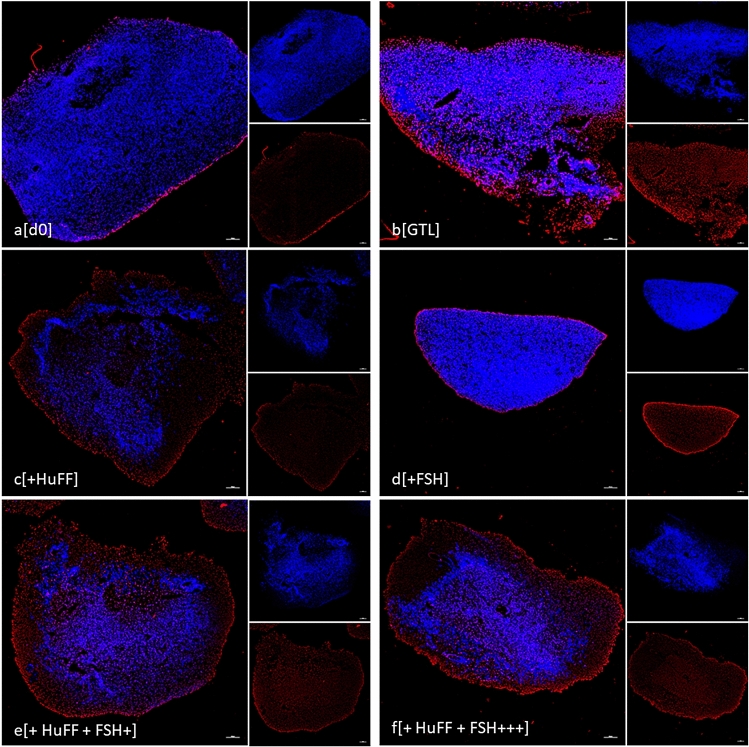
**a**–**f** TUNEL staining of ovarian cortex biopsies. Ovarian cortical tissue was thawed and cultured with the mentioned supplements. After thawing and tissue culture, biopsies were fixed and stained for nicked DNA as a marker of apoptosis induction. Mean staining intensity of whole sections were assessed

**Fig. 7:**
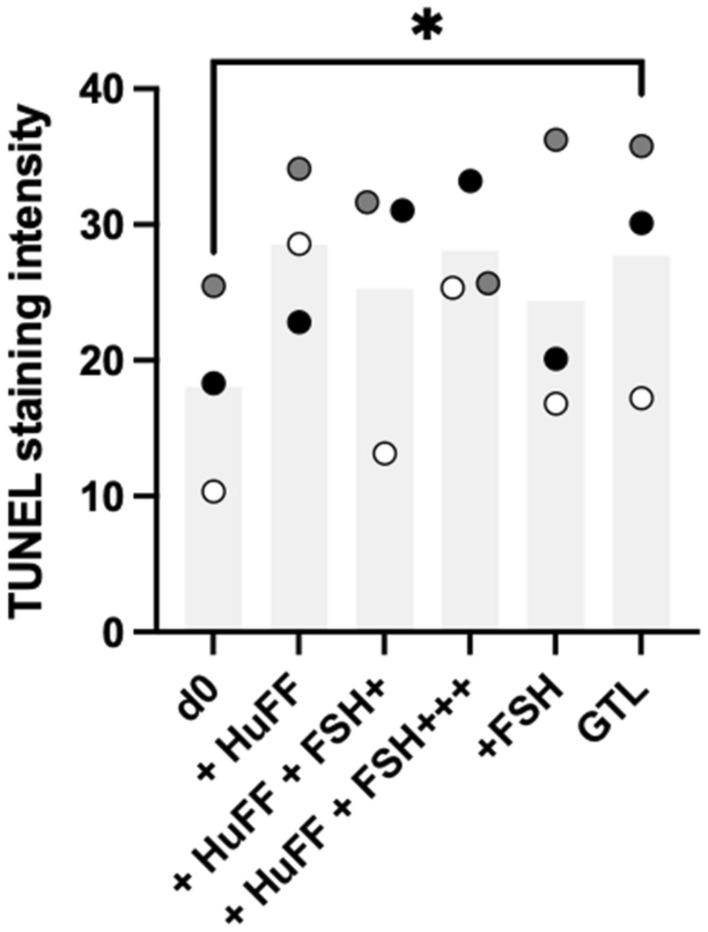
TUNEL staining of ovarian cortex biopsies. Mean TUNEL staining intensity of the tissue section was assessed. Staining control value was subtracted. Each colour represents one patient. Every dot represents the mean of 2 sections of 4 distinct biopsies per patient. Representative fluorescence images from the patient marked filled dots show the TUNEL stain in red and the staining of DNA with DAPI in blue

**Fig. 8:**
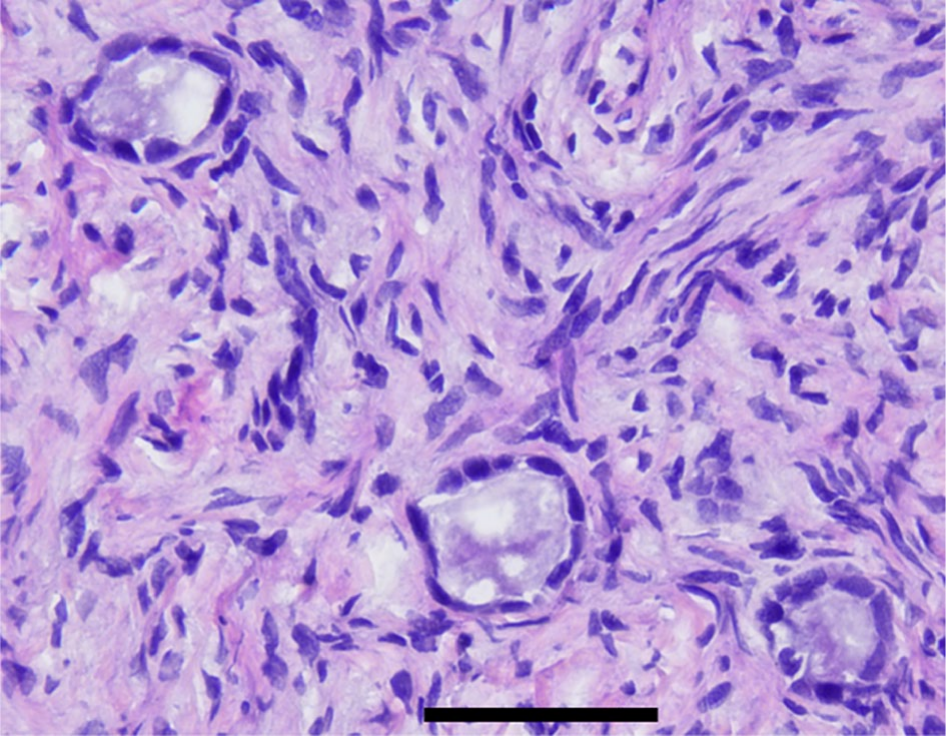
Hematoxylin–Eosin (HE) staining of tissue sections. Complementary histological evaluation of tissue showing the presence of follicles with different sizes. Scalebar 50 µm

Early follicle growth requires tissue integrity. To assess the effect of in vitro culture on apoptosis, tissue was stained for nicked DNA (TUNEL). After thawing, the tissue barely showed sign of apoptosis. After 8 days of culture, the tissue was still viable as seen in the calcein staining. However, the culture in GTL only caused a significant increase in apoptosis. The supplementation of hormones or HuFF showed different patterns in all three patients as indicated in Figs. 6 and 7. Complementary HE staining of tissue sections revealed the presence of follicles with different sizes prior and posterior tissue culture, shown in Fig. 8.

## Discussion

In this study, we first describe the effect of HuFF in a tissue culture system of frozen thawed human ovarian tissue cortex, supplemented with different FSH concentrations under hypoxic conditions.

Analysing the control group on day 1, we found 537 vital follicles with an average size of 39.16 µm—this finding is in agreement with Vanacker et al., observing similar follicle diameters after performing enzymatic isolation ahead of in vitro culture of 7 days—reporting significant growth of follicles but also a substantial loss in follicle numbers [95].

As expected, the majority of vital follicles in the day 1 control group was sized < 50 µm and reached a 87.15% ratio, this also accords with the morphologic properties observed in stained tissue sections, most follicles were primordial and primary stages next to a small proportion of secondary follicles sized > 100 µm. These results are partially consistent with those of Telfer et al. classifying 90% of healthy follicles as primordial or transitory stages when analysing follicular composition prior a tissue culture period of 6 days [96].

In our study, the control group on day 8 (GTL media only) showed significantly smaller vital follicles compared to the day 1 control. As vital follicular stages sized > 100 µm were present in the day 1 control group but not in the day 8 control, these might have become atretic during the culture period. This implicates a certain role of hormones not only for follicular growth but also in maintaining viability of grown follicles.

Treatment group IV (GTL with FSH+++) showed a borderline significance [*P* 0.095] comparing overall vital follicle size with the basis control on day 1. We detected a proportion of follicles sized 50–100 µm of 9.41% while no vital follicles sized > 100 µm were observed. This implicates a non-optimal initiation of follicle activation and a non-sustainment of follicular viability of advanced stages under the used culture conditions. An overall lower FSH concentration and a lack of additional key factors present in HuFF, e.g. activin could have caused this difference to the HuFF-supplemented treatment groups.

Comparing the treatment groups I–III on day 8 with the control on day 1, we observed similar compositions of vital follicles sized > 100 µm but detected significant differences of overall vital follicle size in all 3 groups. On closer examination we detected a considerable increase of vital follicles sized 50–100 µm to 32.64–34.63%, compared to a 11.9% ratio on day 1.

These results partially match those observed in earlier studies of Telfer et al., cultivating ovarian tissue for a period of 6 days with flattened sheets of ovarian cortex [97], while our approach included tissue culture supplementation with HuFF under hypoxic conditions, extensive numeric follicle evaluation via follicular viability tests and large-scale size measurement prior and posterior tissue culture for 8 days.

Furthermore, standardized 2 mm cortex biopsy samples in our culture system offer several advantages: size and numbers of biopsy punches providing a substantial contribution to minimize the effect of unevenly follicle distribution in the human ovary [98–101]. Enhanced nutrition uptake of small cortex samples as in vitro engineered tissues show size dependant constraints due to limited nutrition supply [102, 103]. Additionally, a minimization of the effect of potential follicular interactions in an early or later stage of growth. An extensive appraisal of follicle evaluation increasing the information value regarding follicular size composition of cultured groups as well as conducting viability tests combined with semi-automated size measurements. Finally, 2 mm biopsy punches are well suited for processing in oil overlaid culture drops under hypoxic conditions providing stable and comparable culture conditions similar to IVF embryo culture.

The addition of HuFF (with or without FSH supplementation) did not only hinder the supposed follicular atresia of the day 8 control group, but also promoted follicle growth significantly and sustained follicular viability of larger stages sized > 100 µm of the treatment groups I–III. Given the fact that this size group reached similar proportions as the control group on day 1, it can be assumed that beside viability sustainment a potential growth inhibition of advanced stages occurred that has been described by other groups [104–106].

Results of large-scale viability and size measurements were supplemented by tissue sections, highlighting that enzymatic digestion ahead calcein staining offers a comprehensive approach while tissue sections illuminate solely a sub section of the follicular group composition and may not be representative for the entire cohort, based on that, the majority of biopsy samples were processed for viability testing and calcein staining.

Tissue sections stained for nicked DNA (TUNEL) implicated that the chosen culture conditions predominantly ensured the integrity and viability of the tissue.

As proposed by different working groups, successful IVG of ovarian tissue requires a multi-step [107–110] culture approach, involving follicle activation as a key event in the first step [111, 112]. The mechanisms of follicle activation still need to be elucidated while a combination of maintenance, inhibitory and stimulatory factors is assumed [113], among them activin [114], that is present in follicular fluid [115, 116].

In mammals, the potential of oocyte development [117, 118] is significantly influenced by the follicular environment [119]. According to De Vos et al., when maturating germinal vesicle (GV) oocytes to metaphase II cells (MII) using in vitro maturation (IVM) techniques, fertilization competence depends on the development potential gained under in vivo conditions [120], casting a spotlight on follicular tissue culture mimicking in vivo environment of the early oocyte as best as possible.

Our results demonstrate that tissue culture for 8 days prior follicle isolation offers the advantage of exploiting a high yield of the ovarian follicle reserve through promoting growth initiation while sustaining viability of larger stages simultaneously. This culture approach could facilitate a higher follicular yield while isolating ovarian follicles from unstimulated ovarian tissue bears the risk of obtaining only a small proportion of follicles suited for further single follicle culture. In summary our findings indicate a significant shift from the quiescent to the growing follicle pool while sustaining viability of advanced stages, providing a promising platform for further research in a multi-step culture approach tailored for individual ovarian tissue culture for female cancer patients. We propose that hypoxic ovarian tissue culture supplemented with HuFF and FSH instead of applying single factors like activin or FSH only might benefit IVG of ovarian tissue prior single follicle culture and posterior IVM of GV oocytes as HuFF serves as a rich source of diverse oocyte-nurturing and imprinting factors mimicking in vivo conditions to a large extent.

## Limitations

To maintain protein activity and function, an undamaged sustainment of the 3-D protein structure is obligatory. Batch processing of HuFF included sterile filtration that can have a negative impact on protein quality and quantity [121, 122]. To minimize further negative effects, HuFF samples were frozen in 1 ml aliquots to avoid repeated freezing thawing cycles and stored at − 196 °C in liquid nitrogen to keep protein degradation at a low level. Keeping in mind that HuFF contains more than 800 factors potentially contributing to oocyte growth and development we focussed on the determination of hormonal key factors of the processed batch.

## Data Availability

The data underlying this article will be shared on reasonable request to the corresponding author.

## References

[1] Anderson RA, Amant F, Braat D, D’Angelo A, Susana M, de Sousa C, Lopes ID, Dwek S, Frith L, Lambertini M, Maslin C, Moura-Ramos M, Nogueira D, Rodriguez-Wallberg K, Vermeulen N, The ESHRE Guideline Group on Female Fertility Preservation (2020). ESHRE guideline: female fertility preservation. Hum Reprod Open.

[2] Miller KD, Nogueira L, Mariotto AB, Rowland JH, Robin Yabroff K, Alfano CM, Jemal A, Kramer JL, Siegel RL (2019). Cancer treatment and survivorship statistics. CA Cancer J Clin.

[3] Michalczyk K, Cymbaluk-Płoska A (2021). Fertility preservation and long-term monitoring of gonadotoxicity in girls adolescents and young adults undergoing cancer treatment. Cancers (Basel).

[4] Anderson RA, Clatot F, Demeestere I, Lambertini M, Morgan A, Nelson SM, Cameron FPD (2021). Cancer survivorship: reproductive health outcomes should be included in standard toxicity assessments. Eur J Cancer.

[5] Donnez J, Dolmans M-M (2017). Fertility preservation in women. N Engl J Med.

[6] Jadoul P, Guilmain A, Squifflet J, Luyckx M, Votino R, Wyns C, Dolmans MM (2017). Efficacy of ovarian tissue cryopreservation for fertility preservation: lessons learned from 545 cases. Hum Reprod.

[7] Wallace WH, Kelsey TW, Anderson RA (2016). Fertility preservation in pre-pubertal girls with cancer: the role of ovarian tissue cryopreservation. Fertil Steril.

[8] Kim S, Lee Y, Lee S, Kim T (2018). Ovarian tissue cryopreservation and transplantation in patients with cancer. Obstet Gynecol Sci.

[9] Donnez J, Dolmans MM, Demylle D (2004). Livebirth after orthotopic transplantation of cryopreserved ovarian tissue. Lancet.

[10] Meirow D, Levron J, Eldar-Geva T (2005). Pregnancy after transplantation of cryopreserved ovarian tissue in a patient with ovarian failure after chemotherapy. N Engl J Med.

[11] Donnez J, Dolmans MM, Pellicer A (2015). Fertility preservation for age-related fertility decline. Lancet.

[12] Van der Ven H, Liebenthron J, Beckmann M (2016). Ninety-five orthotopic transplantations in 74 women of ovarian tissue after cytotoxic treatment in a fertility preservation network: tissue activity, pregnancy and delivery rates. Hum Reprod.

[13] Meirow D, Ra’anani H, Shapira M (2016). Transplantations of frozen-thawed ovarian tissue demonstrate high reproductive performance and the need to revise restrictive criteria. Fertil Steril.

[14] Rodriguez-Wallberg KA, Tanbo T, Tinkanen H (2016). Ovarian tissue cryopreservation and transplantation among alternatives for fertility preservation in the Nordic countries—compilation of 20 years of multicenter experience. Acta Obstet Gynecol Scand.

[15] Jensen AK, Macklon KT, Fedder J, Ernst E, Humaidan P, Andersen CY (2017). 86 Successful births and 9 ongoing pregnancies worldwide in women transplanted with frozen-thawed ovarian tissue: focus on birth and perinatal outcome in 40 of these children. J Assist Reprod Genet.

[16] Donnez J, Dolmans M-M (2017). Fertility preservation in women. N Engl J Med.

[17] Hoekman EJ, Louwe LA, Rooijers M, van der Westerlaken LAJ, Klijn NF, Pilgram GSK, de Kroon CD, Hilders CGJM (2019). Ovarian tissue cryopreservation: low usage rates and high live-birth rate after transplantation. Acta Obstet Gynecol Scand.

[18] Reid H, Marsden HB (1980). Gonadal infiltration in children with leukemia and lymphoma. J Clin Pathol.

[19] Bastings L, Beerendonk CCM, Westphal JR, Massuger LFAG, Kaal SEJ, van Leeuwen FE, Braat DDM, Peek R (2013). Autotransplantation of cryopreserved ovarian tissue in cancer survivors and the risk of reintroducing malignancy: a systematic review. Hum Reprod Update.

[20] Shaw JM, Bowles J, Koopman P, Wood EC, Trounson AO (1996). Fresh and cryopreserved ovarian tissue samples from donors with lymphoma transmit the cancer to graft recipients. Hum Reprod.

[21] Meirow D, Hardan I, Dor J, Fridman E, Elizur S, Ra’anani H, Slyusarevsky E, Amariglio N, Schiff E, Rechavi G (2008). Searching for evidence of disease and malignant cell contamination in ovarian tissue stored from hematologic cancer patients. Hum Reprod.

[22] Meirow D (2008). Fertility preservation in cancer patients using stored ovarian tissue: clinical aspects. Curr Opin Endocrinol Diabetes Obes.

[23] Seshadri T, Gook D, Lade S, Spencer A, Grigg A, Tiedemann K, McKendrick J, Mitchell P, Stern C, Seymour JF (2006). Lack of evidence of disease contamination in ovarian tissue harvested for cryopreservation from patients with Hodgkin lymphoma and analysis of factors predictive of oocyte yield. Br J Cancer.

[24] Kristensen SG, Giorgione V, Humaidan P, Alsbjerg B, Bjørn AMB, Ernst E, Andersen CY (2017). Fertility preservation and refreezing of transplanted ovarian tissue—a potential new way of managing patients with low risk of malignant cell recurrence. Fertil Steril.

[25] von Wolff M, Sänger N, Liebenthron J (2018). Is ovarian tissue cryopreservation and transplantation still experimental? It is a matter of female age and type of cancer. J Clin Oncol.

[26] Sanchez-Serrano M, Novella-Maestre E, Rosello-Sastre E, Camarasa N, Teruel J, Pellicer A (2009). Malignant cells are not found in ovarian cortex from breast cancer patients undergoing ovarian cortex cryopreservation. Hum Reprod.

[27] Abir R, Feinmesser M, Yaniv I, Fisch B, Cohen IJ, Ben-Haroush A, Meirow D, Felz C, Avigad S (2010). Occasional involvement of the ovary in Ewing sarcoma. Hum Reprod.

[28] Dolmans MM, Marinescu C, Saussoy P, Van Langendonckt A, Amorim C, Donnez J (2010). Reimplantation of cryopreserved ovarian tissue from patients with acute lymphoblastic leukemia is potentially unsafe. Blood.

[29] Smitz J, Dolmans MM, Donnez J, Fortune JE, Hovatta O, Jewgenow K, Picton HM, Plancha C, Shea LD, Stouffer RL, Telfer EE, Woodruff TK, Zelinski MB (2010). Current achievements and future research directions in ovarian tissue culture, in vitro follicle development and transplantation: implications for fertility preservation. Hum Reprod Update.

[30] Dolmans MM, Martinez-Madrid B, Gadisseux E, Guiot Y, Yuan WY, Torre A, Camboni A, Van Langendonckt A, Donnez J (2007). Short-term transplantation of isolated human ovarian follicles and cortical tissue into nude mice. Reproduction.

[31] Rosendahl M, Andersen MT, Ralfkiær E, Kjeldsen L, Andersen MK, Andersen CY (2010). Evidence of residual disease in cryopreserved ovarian cortex from female patients with leukemia. Fertil Steril.

[32] Vanacker J, Camboni A, Dath C, Van Langendonckt A, Dolmans MM, Donnez J, Amorim CA (2011). Enzymatic isolation of human primordial and primary ovarian follicles with Liberase DH: protocol for application in a clinical setting. Fertil Steril.

[33] Soares M, Saussoy P, Maskens M, Reul H, Amorim CA, Donnez J, Dolmans M-M (2017). Eliminating malignant cells from cryopreserved ovarian tissue is possible in leukaemia patients. Br J Haematol.

[34] Soares M, Sahrari K, Amorim CA, Saussoy P, Donnez J, Dolmans MM (2015). Evaluation of a human ovarian follicle isolation technique to obtain disease-free follicle suspensions before safely grafting to cancer patients. Fertil Steril.

[35] Mouloungui E, Zver T, Roux C, Amiot C (2018). A protocol to isolate and qualify purified human preantral follicles in cases of acute leukemia, for future clinical applications. J Ovarian Res.

[36] Gaixia Xu, Lin S, Law W-C, Roy I, Lin X, Mei S, Ma H, Chen S, Niu H, Wang X (2012). The invasion and reproductive toxicity of QDs-transferrin bioconjugateson preantral folliclein vitro. Theranostics.

[37] Mitchell LM, Kennedy CR, Hartshorne GM (2002). Effects of varying gonadotrophin dose and timing on antrum formation and ovulation effciency of mouse follicles in vitro. Hum Reprod.

[38] Jee BC, Kim JH, Park DH, Youm H, Suh CS, Kim SH (2012). In vitro growth of mouse preantral follicles: effect of animal age and stem cell factor/insulin-like growth factor supplementation. Clin Exp Reprod Med.

[39] Choi JH, Yoo CR, Ahn JY, Park JH, Lim JM (2012). Growth of ovarian primary follicles retrieved from neonates of different ages and derivation of mature oocytes following in vitro-culture. Asian-Australas J Anim Sci.

[40] Smitz J, Dolmans MM, Donnez J, Fortune JE, Hovatta O, Jewgenow K, Picton HM, Plancha C, Shea LD, Stouffer RL, Telfer EE, Woodruff TK, Zelinski MB (2010). Current achievements and future research directions in ovarian tissue culture, in vitro follicle development and transplantation: implications for fertility preservation. Hum Reprod Update.

[41] Eppig JJ, O’Brien MJ (1996). Development in-vitro of mouse oocytes from primordial follicles. Biol Reprod.

[42] Lenie S, Cortvrindt R, Adriaenssens T, Smitz J (2004). A reproducible two-step culture system for isolated primary mouse ovarian follicles as single functional units. Biol Reprod.

[43] Smitz J, Cortvrindt R (2002). The earliest stages of folliculogenesis in-vitro. Reproduction.

[44] Wandji S-A, Eppig JJ, Fortune JE (1996). FSH and growth factors affect the growth and endocrine function in-vitro of granulosa cells of bovine preantral follicles. Theriogenology.

[45] Smitz J, Dolmans MM, Donnez J, Fortune JE, Hovatta O, Jewgenow K, Picton HM, Plancha C, Shea LD, Stouffer RL, Telfer EE, Woodruff TK, Zelinski MB (2010). Current achievements and future research directions in ovarian tissue culture, in vitro follicle development and transplantation: implications for fertility preservation. Hum Reprod Update.

[46] McLaughlin M, Albertini DF, Wallace WHB, Anderson RA, Telfer EE (2018). Metaphase II oocytes from human unilaminar follicles grown in a multi-step culture system. Mol Hum Reprod.

[47] Anckaert E, De Rycke M, Smitz J (2013). Culture of oocytes and risk of imprinting defects. Hum Reprod Update.

[48] Telfer EE (2019). Future developments: in vitro growth (IVG) of human ovarian follicles. Acta Obstet Gynecol Scand.

[49] Lewandowska AE, Macur K, Czaplewska P, Liss J, Łukaszuk K, Ołdziej S (2017). Qualitative and quantitative analysis of proteome and peptidome of human follicular fluid using multiple samples from single donor with LC−MS and SWATH methodology. J Proteome Res.

[50] Zamah AM, Hassis ME, Albertolle ME, Williams KE (2015). Proteomic analysis of human follicular fluid from fertile women. Clin Proteomics.

[51] Chen F, Spiessens C, D’Hooghe T, Peeraer K, Carpentier S (2016). Follicular fluid biomarkers for human in vitro fertilization outcome: proof of principle. Proteome Sci.

[52] Ambekar AS, Kelkar DS, Pinto SM, Sharma R, Hinduja I, Zaveri K, Pandey A, Prasad TSK, Gowda H, Mukherjee S (2015). Proteomics of follicular fluid from women with polycystic ovary syndrome suggests molecular defects in follicular development. J Clin Endocrinol Metab.

[53] Kushnir MM, Naessén T, Wanggren K, Rockwood AL, Crockett DK, Bergquist J (2012). Protein and steroid profiles in follicular fluid after ovarian hyperstimulation as potential biomarkers of IVF outcome. J Proteome Res.

[54] Hanrieder J, Zuberovic A, Bergquist J (2009). Surface modified capillary electrophoresis combined with in solution isoelectric focusing and MALDI-TOF/TOF MS: a gel-free multidimensional electrophoresis approach for proteomic profiling-exemplified on human follicular fluid. J Chromatogr A.

[55] Bianchi L, Gagliardi A, Campanella G, Landi C, Capaldo A, Carleo A, Armini A, De Leo V, Piomboni P, Focarelli R (2013). A methodological and functional proteomic approach of human follicular fluid en route for oocyte quality evaluation. J Proteomics.

[56] Ambekar AS, Nirujogi RS, Srikanth SM, Chavan S, Kelkar DS, Hinduja I, Zaveri K, Prasad TSK, Harsha HC, Pandey A (2013). Proteomic analysis of human follicular fluid: a new perspective towards understanding folliculogenesis. J Proteomics.

[57] Regiani T, Cordeiro FB, da Costa L, Salgueiro J, Cardozo K, Carvalho VM, Perkel KJ, Zylbersztejn DS, Cedenho AP, Lo Turco EG (2015). Follicular fluid alterations in endometriosis: label-free proteomics by MS(E) as a functional tool for endometriosis. Syst Biol Reprod Med.

[58] Lo Turco EG, Cordeiro FB, de Carvalho Lopes PH, Gozzo FC, Pilau EJ, Soler TB, da Silva BF, Del Giudice PT, Bertolla RP, Fraietta R (2013). Proteomic analysis of follicular fluid from women with and without endometriosis: new therapeutic targets and biomarkers. Mol Reprod Dev.

[59] Yoo SW, Bolbot T, Koulova A, Sneeringer R, Humm K, Dagon Y, Usheva A (2013). Complement factors are secreted in human follicular fluid by granulosa cells and are possible oocyte maturation factors. J Obstet Gynaecol Res.

[60] Hanrieder J, Nyakas A, Naessén T, Bergquist J (2008). Proteomic analysis of human follicular fluid using an alternative bottom-up approach. J Proteome Res.

[61] Schweigert FJ, Gericke B, Wolfram W, Kaisers U, Dudenhausen JW (2006). Peptide and protein profiles in serum and follicular fluid of women undergoing IVF. Hum Reprod.

[62] Wu Y-T, Wu Y, Zhang J-Y, Hou N-N, Liu A-X, Pan J-X, Lu J-Y, Sheng J-Z, Huang H-F (2015). Preliminary proteomic analysis on the alterations in follicular fluid proteins from women undergoing natural cycles or controlled ovarian hyperstimulation. J Assist Reprod Genet.

[63] Liu X, Wang Y, Zhu P, Wang J, Liu J, Li N, Wang W, Zhang W, Zhang C, Wang Y, Shen X, Liu F (2020). Human follicular fluid proteome reveals association between overweight status and oocyte maturation abnormality. Clin Proteom.

[64] Zamah AM, Hassis ME, Albertolle ME, Williams KE (2015). Proteomic analysis of human follicular fluid from fertile women. Clin Proteomics.

[65] Xia L, Zhao X, Sun Y, Hong Y, Gao Y, Hu S (2014). Metabolomic profiling of human follicular fluid from patients with repeated failure of in vitro fertilization using gas chromatography/mass spectrometry. Int J Clin Exp Pathol.

[66] Revelli A, Piane LD, Casano S, Molinari E, Massobrio M, Rinaudo P (2009). Follicular fluid content and oocyte quality: from single biochemical markers to metabolomics. Reprod Biol Endocrinol.

[67] Emori MM, Drapkin R (2014). The hormonal composition of follicular fluid and its implications for ovarian cancer pathogenesis. Reprod Biol Endocrinol.

[68] de Santoslos MJ, Garcıa-Laez V, Beltran D, Labarta E, Zuzuarregui JL, Alama P, Gamiz P, Crespo J, Bosch E, Pellicer A (2012). The follicular hormonal profile in low-responder patients undergoing unstimulated cycles: is it hypoandrogenic?. Hum Reprod.

[69] Zamah AM, Hassis ME, Albertolle ME, Williams KE (2015). Proteomic analysis of human follicular fluid from fertile women. Clin Proteomics.

[70] Revelli A, Piane LD, Casano S, Molinari E, Massobrio M, Rinaudo P (2009). Follicular fluid content and oocyte quality: from single biochemical markers to metabolomics. Reprod Biol Endocrinol.

[71] Piccinni M-P, Vicenti R, Logiodice F, Fabbri R, Kullolli O, Pallecchi M, Paradisi R, Danza G, Macciocca M, Lombardelli L, Seracchioli R (2021). Description of the follicular fluid cytokine and hormone profiles in human physiological natural cycles. J Clin Endocrinol Metab.

[72] Bianchi L, Gagliardi A, Landi C, Focarelli R, de leo V, Luddi A, Bini L, Piomboni P (2016). Protein pathways working in human follicular fluid: the future for tailored IVF?. Expert Rev Mol Med.

[73] Lewandowska EA, Macur K, Czaplewska P, Liss J, Łukaszuk K, Ołdziej S (2017). Qualitative and quantitative analysis of proteome and peptidome of human follicular fluid using multiple samples from single donor with LC−MS and SWATH methodology. J Proteome Res.

[74] Molaeeghaleh N, Tork S, Abdi S, Movassaghi S (2020). Evaluating the effects of different concentrations of human follicular fluid on growth, development, and PCNA gene expression of mouse ovarian follicles. Cells Tissues Organs.

[75] Ng KYB, Mingels R, Morgan H, Macklon N, Cheong Y (2018). In vivo oxygen, temperature and pH dynamics in the female reproductive tract and their importance in human conception: a systematic review. Hum Reprod Update.

[76] von Mengden L, Klamt F, Smitz J (2020). Redox biology of human cumulus cells: basic concepts, impact on oocyte quality, and potential clinical use. Antioxid Redox Signal.

[77] Lim M, Thompson JG, Dunning KR (2021). Hypoxia and ovarian function: follicle development, ovulation, oocyte maturation. Reproduction.

[78] Gook DA, Edgar DH, Lewis K, Sheedy JR, Gardner DK (2014). Molecular impact of oxygen concentration on adult murine pre-antral follicle development in vitro and the corresponding metabolic profile. Hum Reprod.

[79] Eppig JJ, Wigglesworth K (1995). Factors affecting the developmental competence of mouse oocytes grown in vitro: oxygen concentration. Mol Reprod Dev.

[80] Gook DA, Edgar DH, Lewis K, Sheedy JR, Gardner DK (2014). Impact of oxygen concentration on adult murine pre-antral follicle development in vitro and the corresponding metabolic profile. Mol Hum Reprod.

[81] Gook DA, Edgar DH, Lewis K, Sheedy JR, Gardner DK (2014). Impact of oxygen concentration on adult murine pre-antral follicle development in vitro and the corresponding metabolic profile. Mol Hum Reprod.

[82] Heise MK, Koepsel R, McGee EA, Russell AJ (2009). Dynamic oxygen enhances oocyte maturation in long-term follicle culture. Tissue Eng Part C.

[83] Gosden RG, Baird DT, Wade JC, Webb R (1994). Restoration of fertility to oophorectomized sheep by ovarian autografts stored at −196 °C. Hum Reprod.

[84] Isachenko V, Isachenko E, Reinsberg J, Montag M, van der Ven K, Dorn C, Roesing B, van der Ven H (2007). Cryopreservation of human ovarian tissue: comparison of rapid and conventional freezing. Cryobiology.

[85] Fabbri R, Venturoli S, D’Errico A, Iannascoli C, Gabusi E, Valeri B, Seracchioli R, Grigioni WF (2003). Ovarian tissue banking and fertility preservation in cancer patients: histological and immunohistochemical evaluation. Gynecol Oncol.

[86] Martinez-Madrid B, Dolmans M-M, Van Langendonckt A, Defrère S, Donnez J (2004). Fertil Steril.

[87] Leonel ECR, Lucci CM, Amorim CA (2019). Cryopreservation of human ovarian tissue: a review. Transfus Med Hemother.

[88] Liebenthron J, Montag M, Reinsberg J, Köster M, Isachenko V, van der Ven K, van der Ven H, Krüssel J-S, von Wolff M (2019). Overnight ovarian tissue transportation for centralized cryobanking: a feasible option. RBMO.

[89] European Union (2004) Directive 2004/23/EC of the European Parliament and of the Council of 31 March 2004 on setting standards of quality and safety for the donation, procurement, testing, processing, preservation, storage, and distribution of human tissues and cells. Off J Eur Un, L 102/48.

[90] Mortimer D (2005). A critical assessment of the impact of the European union tissues and cells directive (2004) on laboratory practices in assisted conception. Reprod Biomed Online.

[91] Ryu AH, Eckalbar WL, Kreimer A, Yosef N, Ahituv N (2017). Use antibiotics in cell culture with caution: genome-wide identifcation of antibiotic-induced changes in gene expression and regulation. Sci Rep.

[92] Skubis A, Gola J, Sikora B, Hybiak J, Paul-Samojedny M, Mazurek U, Łos MJ (2017). Impact of antibiotics on the proliferation and differentiation of human adipose-derived mesenchymal stem cells. Int J Mol Sci.

[93] Cortvrindt Rita G, Smitz Johan E. J (2001). Fluorescent probes allow rapid and precise recording of follicle density and staging in human ovarian cortical biopsy samples. Fertil Steril.

[94] Kristensen SG, Ebbesen P, Andersen CY (2015). Transcriptional profiling of five isolated size-matched stages of human preantral follicles. Mol Cell Endocrinol.

[95] Vanacker J, Camboni A, Dath C, Van Langendonckt A, Dolmans M-M, Donnez J, Amorim CA (2011). Enzymatic isolation of human primordial and primary ovarian follicles with Liberase DH: protocol for application in a clinical setting. Fertil Steril.

[96] Telfer EE, McLaughlin M, Christina DK, Thong J (2008). A two-step serum-free culture system supports development of human oocytes from primordial follicles in the presence of activin. Hum Reprod.

[97] Telfer EE, McLaughlin M, Christina DK, Thong J (2008). A two-step serum-free culture system supports development of human oocytes from primordial follicles in the presence of activin. Hum Reprod.

[98] Schmidt KLT, Byskov AG, Andersen AN, Müller J, Andersen CY (2003). Density and distribution of primordial follicles in single pieces of cortex from 21 patients and in individual pieces of cortex from three entire human ovaries. Hum Reprod.

[99] Poirot C, Vacher-Lavenu M-C, Helardot P, Guibert J, BrugieÁres L, Jouannet P (2002). Human ovarian tissue cryopreservation: indications and feasibility. Hum Reprod.

[100] Qu J, Godin PA, Nisolle M, Donnez J (2000). Distribution and epidermal growth factor receptor expression of primordial follicles in human ovarian tissue before and after cryopreservation. Hum Reprod.

[101] Kohl J, Dittrich R, Siebzehnrübl E, Wildt L (2000). Determination of follicle numbers in human ovarian biopsies ± a method for estimation of outcome of ovarian cryopreservation. Fertil. Steril.

[102] Griffith LG, Naughton G (2002). Tissue engineering—current challenges and expanding opportunities. Science.

[103] Rademakers T, Horvath JM, van Blitterswijk CA, Vanessa LS (2019). LaPointe oxygen and nutrient delivery in tissue engineering: approaches to graft vascularization. J Tissue Eng Regen Med.

[104] Hovatta O, Wright C, Krausz T, Hardy K, Winston RM (1999). Human primordial, primary and secondary ovarian follicles in long-term culture: effect of partial isolation. Hum Reprod.

[105] Telfer EE, McLaughlin M, Ding C, Thong KJ (2008). A two-step serumfree culture system supports development of human oocytes from primordial follicles in the presence of activin. Hum Reprod.

[106] Telfer EE (2019). Future developments: in vitro growth (IVG) of human ovarian follicles. Acta Obstet Gynecol Scand.

[107] Telfer EE (2019). Future developments: in vitro growth (IVG) of human ovarian follicles. Acta Obstet Gynecol Scand.

[108] Telfer EE, McLaughlin M, Ding C, Thong KJ (2008). A two-step serumfree culture system supports development of human oocytes from primordial follicles in the presence of activin. Hum Reprod.

[109] McLaughlin M, Albertini DF, Wallace WHB, Anderson RA, Telfer EE (2018). Metaphase II oocytes from human unilaminar follicles grown in a multi-step culture system. Mol Hum Reprod.

[110] Smitz J, Dolmans MM, Donnez J (2010). Current achievements and future research directions in ovarian tissue culture, in vitro follicle development and transplantation: implications for fertility preservation. Hum Reprod Update.

[111] Telfer EE, McLaughlin M, Ding C, Thong KJ (2008). A two-step serumfree culture system supports development of human oocytes from primordial follicles in the presence of activin. Hum Reprod.

[112] McLaughlin M, Albertini DF, Wallace WHB, Anderson RA, Telfer EE (2018). Metaphase II oocytes from human unilaminar follicles grown in a multi-step culture system. Mol Hum Reprod.

[113] Nelson SM, Telfer EE, Anderson RA (2013). The ageing ovary and uterus: new biological insights. Hum Reprod Update.

[114] Telfer EE, McLaughlin M, Ding C, Thong KJ (2008). A two-step serumfree culture system supports development of human oocytes from primordial follicles in the presence of activin. Hum Reprod.

[115] Lau CP, Ledger WL, Groome NP, Barlow DH, Muttukrishna S (1999). Dimeric inhibins and activin A in human follicular fluid and oocyte-cumulus culture medium. Hum Reprod.

[116] Cupisti S, Dittrich R, Mueller A, Strick R, Stiegler E, Binder H, Beckmann MW, Strissel P (2007). Correlations between anti-müllerian hormone, inhibin B, and activin A in follicular fluid in IVF/ICSI patients for assessing the maturation and developmental potential of oocytes. Eur J Med Res.

[117] Thompson JG, Gilchrist RB, Trounson GR, Eichenlaub-Ritter U (2013). Improving oocyte maturation in vitro. Biology and Pathology of the oocyte: role in fertility, medicine, and nuclear reprogramming.

[118] Luciano AM, Sirard MA (2018). Successful in vitro maturation of oocytes: a matter of follicular differentiation. Biola Reprod.

[119] De Vos M, Grynberg M, Ho TM, Yuan Ye, Albertini DF, Gilchrist RB (2021). Perspectives on the development and future of oocyte IVM in clinical practice. J Assist Reprod Genet.

[120] De Vos M, Grynberg M, Ho TM, Yuan Ye, Albertini DF, Gilchrist RB (2021). Perspectives on the development and future of oocyte IVM in clinical practice. J Assist Reprod Genet.

[121] Maa YF, Hsu CC (1998). Investigation of fouling mechanisms for recombinant human growth hormone sterile filtration. J Pharm Sci.

[122] van den Oetelaar PJM, Mentink IM, Brinks GJ (1989). Loss of peptides and proteins upon sterile filtration due to adsorption to membrane filters. Drug Dev Ind Pharm.

